# Taxonomic updates in *Amphitecna* (Bignoniaceae): A new Mexican species and the re-establishment of the giant-leaved *A. megalophylla*

**DOI:** 10.3897/phytokeys.171.55397

**Published:** 2021-01-25

**Authors:** Héctor Gómez-Domínguez, Andrés Ernesto Ortiz-Rodríguez, Delfilia Velasco-Espino, Rene Hernández-Burguete

**Affiliations:** 1 Herbario Eizi Matuda (HEM) Instituto de Ciencias Biológicas, Universidad de Ciencias y Artes de Chiapas, Tuxtla Gutiérrez, Chiapas, Mexico Universidad de Ciencias y Artes de Chiapas Tuxtla Gutiérrez Mexico; 2 Departamento de Botánica, Instituto de Biología, Universidad Nacional Autónoma de México, Mexico Universidad Nacional Autónoma de México México Mexico

**Keywords:** Chiapas, conservation, Crescentieae, Neotropics, Mexico

## Abstract

In this study, we analyzed the morphological affinities of the 24 species of *Amphitecna* based on detailed morphological studies and multivariate cluster analyses. Our results suggest that the genus *Amphitecna* includes six morphological groups that can be easily distinguished based on floral and fruits characteristics: *A. donnell-smithii* group, *A. macrophylla* group, *A. megalophylla* group, *A. molinae* group, *A. spathicalyx* group, and *A. steyermarkii* group. A new species from Mexico, *Amphitecna fonceti*, is described. This new species is clearly differentiated by the predominantly ramiflorous inflorescences bearing multiple flowers per shoot, buds rounded at the apex, large flowers with a transverse fold in the corolla throat, calyx surface pubescent and strongly costate, and fruits elliptic, apiculate at the apex. We discuss the characteristics of each morphological group and their geographical distribution, provide a detailed description of the new species including ethnobotany notes, and propose the re-establishment of the giant-leaved species *A. megalophylla*.

## Introduction

*Amphitecna* Miers is a Neotropical genus of small to medium-size trees (Gentry 1980). The genus includes around 25 species, most restricted to tropical rainforests. Molecular phylogenetic and morphological data indicate that *Amphitecna* is closely related to the Neotropical genera *Crescentia* L. and *Parmentiera* DC., from which it differs by the combination of simple and alternate leaves, greenish corollas with petal lobes fused, and pepo-type fruits (Gentry 1980, Grose and Olmstead 2007).

The distribution range of *Amphitecna* encompasses two regions with high species diversity (Table 1). The first region includes 14 species and encompasses the rainforests from Mexico to Honduras. The second region contains eight endemic species that range from Northern Nicaragua to the North of Colombia (Table 1). The only widely distributed species is *A. latifolia* (Mill.) A.H. Gentry, that occurs in the coastal areas of Florida (USA), the Caribbean, the Pacific slope of Mexico, and coastal areas of Central America to Colombia, Ecuador, and Venezuela (Gentry 1980). The wide distribution of *A. latifolia* may be associated with its water-dispersed fruits, while most of the narrowly distributed species are mammal-dispersed (Gentry 1980).

Reproductive characteristics of species of *Amphitecna* are quite variable, with most species exhibiting clear differences in flower and fruit morphology (Gentry 1980; Gentry 1982a; Ortiz-Rodriguez et al. 2016). However, most species are similar vegetatively, often leading to taxonomic confusion and misidentifications. For example, the four giant-leaved species, with leaves 50–100 cm long × 10–15 cm wide, i.e., *Amphitecna costata*, *A. megalophylla*, *A. macrophylla*, and *A. regalis*, are frequently misidentified in herbaria. Despite the similarity in leaf traits, these four species are easily differentiated reproductively (Gentry 1980, Table 2). Among the most variable reproductive features of *Amphitecna* are the corolla shape, inflorescence position, number of flowers per shoot, pedicel length, and the number of calyx components (Gentry 1980; Gentry 1982a; Burger and Gentry 2000). Flower bud shape, fruit morphology, and calyx surface also represent important features for species identification (Ortiz-Rodriguez et al. 2016).

Here, we carried out a multivariate cluster analyses of all 24 species of *Amphitecna* currently recognized to infer the morphological affinities among species and establish the position of a newly described species of *Amphitecna* from the Sierra Madre de Chiapas, Mexico.

## Materials and methods

To infer the morphological similarities among the 24 species of *Amphitecna*, we performed a hierarchical clustering analysis on a matrix that included 15 flower traits. The data were analyzed using the unweighted pair group method with arithmetic mean (UPGMA, Sokal and Michener 1958) and the Gower index (Gower 1971), allowing a simultaneous use of binary and continuous characters (Dunn and Everitt 1982; Yang et al. 2007; Zanella et al. 2011; Tuler et al. 2017; Svoboda and Ballard 2018; Wahlsteen and Tyler 2019). All morphological characters were obtained from original species descriptions (Gentry 1980; Gentry 1982b; Burger and Gentry 2000; Ortiz-Rodriguez et al. 2016), herbarium specimens deposited at MEXU (www.ibdata.ib.unam.mx), and type specimens available online (https://www.gbif.org/ and https://plants.jstor.org/).

The UPGMA results were contrasted with those derived from other clustering algorithms, specifically Ward, single linkage, complete linkage, WPGMA, WPGMC, and UPGMC, implemented in the R-package *stats*, using the function *hclust* (R Core Team 2020: https://www.r-project.org/). We then determined the similarities and differences among the various clustering dendrograms by calculating the cophenetic correlation (a Pearson’s measure) between each clustering result using the *cor.dendlist* and the *corrplot* functions from the *corrplot* R-package (Wei and Simko 2017). For each dendrogram, the agglomerative coefficient was calculated using the *agnes* function from the *cluster* R-package (Maechler et al. 2019). The agglomerative coefficient measures the amount of clustering structure, with values closer to 1 suggesting stronger clustering structure. Also, the Fowlkes-Mallows Index (from the *dendextend* R-package, Galili 2015) was used to compare the species composition within clusters (k = 3–8) obtained from the UPGMA analysis and other algorithms. The optimal number of morphological clusters in *Amphitecna* was determined based on the greater similarity between clustering algorithms (values closer to 1). We further performed an internal clustering validation (a cluster stability test) by calculating the average silhouette width (*Si*) for each cluster (k = 3–8) resulting from each of the algorithms used. While *Si* values greater than 0.71 suggest strong structure and cluster stability, values between 0.51 and 0.70 are interpreted as reasonable, values between 0.26 and 0.50 indicate weak structure, and values lower than or equal to 0.25 are not worth further discussion (Kaufman and Rousseeuw 2005). The graphical representation of the UPGMA dendrogram was carried out in the R software, using the function *hclust* implemented in the R-packages *ape*, and *ggtree* (Yu et al. 2017; Paradis and Schliep 2018).

The new species described was recognized by a unique combination of features (Donoghue 1985) identified through comparisons with morphologically similar taxa and literature review (Gentry 1982a, b, Burger and Gentry 2000; Ortiz-Rodriguez et al. 2016). We assessed the conservation status by calculating the extent of occurrence (EOO) and the area of occupancy (AOO) using the GeoCAT tool (Bachman et al. 2011) and applying the IUCN Red List Categories and criteria (IUCN 2019).

## Results

**Figure 1.**
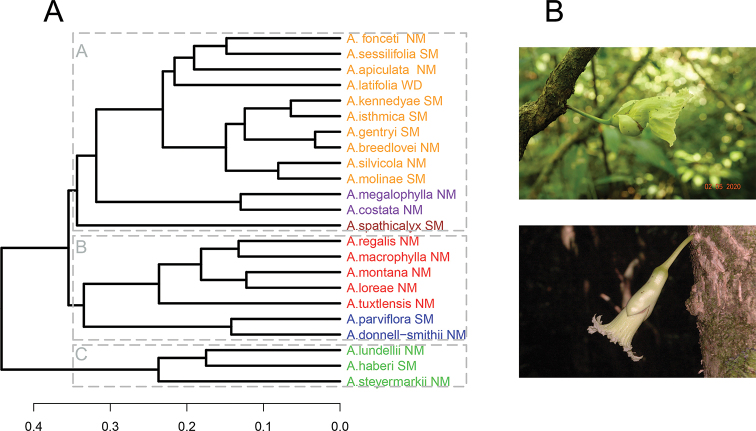
Morphological similarities among the 24 species of *Amphitecna* currently recognized **A** dendrogram based on the results from an UPGMA analysis **B** main flower types found in *Amphitecna*, flowers with a transverse fold in the corolla throat (*Amphitecna costata*, top right) and flowers radially symmetric, without a transverse fold in the throat (*Amphitecna tuxtlensis*, bottom right). Photographs by Hector Gómez Domínguez (*A. costata*) and Pablo Carrillo Reyes (*A. tuxtlensis*). NM = Northern Mesoamerica; SM = Southern Mesoamerica.

The UPGMA dendrogram is shown in Figure 1. The results of this analysis are very similar to those obtained using other clustering algorithms (correlation values between 0.78 and 0.98, Suppl. material 1: Figure S1). The agglomerative coefficient value for the UPGMA dendrogram was 0.67 (between 0.47 and 0.83 in analyses conducted with other approaches), suggesting a moderate to strong structure among species of *Amphitecna*. The Fowlkes-Mallows Index showed that six groups (k = 6) show significantly similar clusters when the UPGMA is compared to the other clustering algorithms (FM values between 0.71 and 1). Silhouette width values consistently showed the highest values (*Si* value 0.35 for all algorithms) when each dendrogram was divided into six clusters.

The UPGMA results indicate that the genus *Amphitecna* can be classified into six morphological groups (Figure 1A) and three main clusters (A–C). The *A. molinae*, *A. megalophylla*, and *A. spathicalyx* groups are characterized by corollas with a transverse fold in the throat (Figure 1B). In the UPGMA dendrogram, these species are nested within cluster “A” (Figure 1A). The *Amphitecna molinae* group consists of 10 species with sessile leaves, 15–40 cm long, flower buds rounded at apex, and smooth fruit surface, rarely warty. The *A. megalophylla* group includes two species with short-petiolate leaves, long leaf blades (50–100 cm long), flower buds rounded at the apex, and fruit surface costate. The new species, *Amphitecna fonceti*, is part of the *A. molinae* group and is morphologically most similar to the *A. latifolia* and *A. sessilifolia* groups (Table 3). *Amphitecna spathicalyx* is also placed within cluster “A” (i.e., the *A. spathicalyx* group) and is distinguished from other species by the pointed flower buds and spathaceous calyces.

Cluster “B” is composed of the *A. macrophylla* and *A. donnell-smithii* groups (Figure 1A). The species from these groups are best recognized by their short-petiolate leaves, inflorescences born along the main trunk or leafless branches, mostly composed of 1–2 flowers per shoot, flowers without a transverse fold in the corolla throat, and calyces bilabiate or trilabiate (Figure 1B). The *A. macrophylla* group contains five species with cauliflorous inflorescences and long and funnelform corollas (up to 70 mm long). The *A. donnell-smithii* group consists of two ramiflorous species with small flowers (less than 28 mm long) and broadly campanulate corollas. The only two species of *Amphitecna* with broadly campanulate flowers are included in the *A. donnell-smithii* group.

Cluster “C” consists of the *Amphitecna steyermarkii* group and is composed of three species that are characterized by their terminal inflorescences with several flowers per shoot, flower buds with a sharp acumen, flowers without a transverse fold in the corolla throat, and spathaceous calyces.

### Key for the identification of species of *Amphitecna* (Bignoniaceae)

**Table:** 

1	Corollas with a transverse fold in the throat	**2**
–	Corollas without a transverse fold in the throat	**3**
2	Flowers buds with a sharp acumen and spathaceous calyx	***A. spathicalyx* (Panama and Colombia)**
–	Flowers buds obtuse to rounded at apex (rarely acute in *A. sessilifolia*); calyx bilabiate or trilabiate	**4**
3	Inflorescences terminal; flowers buds with a sharp acumen (sometimes lacking in *A. haberi*)	**5**
–	Inflorescences on leafless portions of branches and throughout the main trunk; flower buds obtuse to rounded at apex (acute in *A. tuxtlensis*)	**6**
4	Inflorescences terminal	**7**
–	Inflorescences axillary, on leafless portions of branches and throughout the main trunk	**8**
5	Leaves shortly petiolate, up to 18 cm long, acute at the base; longer pedicels up to 75 mm long	**9**
–	Leaves sessile, often longer than 20 cm long, obtuse to rounded at base; longer pedicels up to 50 mm long	***A. steyermarkii* (Mexico and Guatemala)**
6	Pedicels 60–100 mm long; fruits globose or nearly so, rounded at apex	**10**
–	Pedicels 10–40 mm long; fruits elliptic to narrowly elliptic, acute to apiculate at apex	**11**
7	Inflorescences composed of 3-to-several flowers per shoot	**12**
–	Inflorescences composed of 1 or 2 flowers per shoot	**13**
8	Pachycaul trees; leaves 50–100 cm long; fruit surface costate	**14**
–	Branched trees; leaves 15–40 cm long; fruit surface smooth or rough, rarely costate	**15**
9	Inflorescences composed of 1 or 2 flowers per shoot; calyx spathaceous, up to 45 mm long	***Amphitecna lundellii* (Guatemala and Belize)**
–	Inflorescences composed of 3-to-several flowers per shoot; calyx bilabiate, up to 20 mm long	***Amphitecna haberi* (Costa Rica)**
10	Mature leaves longer than 25 cm; inflorescences ramiflorous, composed of one or two flowers per shoot; pedicels more than 70 mm long	***A. montana* (Mexico and Guatemala)**
–	Mature leaves shorter than 25 cm long; inflorescences trunciflorous, composed of 3-to-several flowers per shoot; pedicels less than 70 mm long	***A. loreae* (Mexico)**
11	Pachycaul trees; leaves 50–100 cm long	**16**
–	Branched trees; leaves 18–25 cm long	**17**
12	Corolla tubular, up to 30 mm long; calyx almost as long as the corolla	***A. apiculata* (Mexico, Guatemala, and Belize)**
–	Corolla funnelform, up to 65 mm long; calyx much smaller than the corolla	**18**
13	Leaves with 14 secondary veins or more; mature fruits longer than 10 cm long	**19**
–	Leaves with fewer than 14 secondary veins; mature fruits shorter than 10 cm long	**20**
14	Inflorescences composed of 1 or 2 flowers per shoot; pedicels up to 25 mm long	***A. costata* (Guatemala)**
–	Inflorescences composed of 3-to-several flowers per shoot; pedicels up to 60 mm long	***A. megalophylla* (Guatemala)**
15	Leaves shortly petiolate; inflorescences composed of 1 or 2 flowers per shoot; calyx smooth	**21**
–	Leaves sessile; inflorescences composed of 3-to-several flowers per shoot; calyx costate, with 6–10 longitudinal ridges per lobe, surface densely covered by lenticels-like white dots	***A. fonceti* (Mexico)**
16.	Longer leaves up to 100 cm long; pedicels ca. 10 mm long; corolla 52–65 mm long, 23–30 mm wide at mouth of tube	***A. regalis* (Mexico)**
–	Longer leaves up to 60 cm long; pedicels 15–40 mm long; corolla 37–50 mm long, 10–15 mm wide at mouth of tube	***A. macrophylla* (Mexico)**
17	Flower bud rounded at apex; corolla campanulate, up to 30 mm long	**22**
–	Flower buds acute at apex; corolla funnelform, up to 60 mm long	***A. tuxtlensis* (Mexico)**
18	Leaves elliptic or widely obovate, coriaceous; pedicels up to 40 mm long; fruits globose with rounded apex; restricted to coastal ecosystems	***A. latifolia* (widely distributed)**
–	Leaves narrowly obovate to oblanceolate, chartaceous; pedicels up to 80 mm long; fruits elliptic with elongated apex; restricted to montane ecosystems (1300–2000 m alt)	***A. sessilifolia* (Costa Rica and Panama)**
19	Corolla 44–60 mm long	***A. isthmica* (Costa Rica and Panama)**
–	Corolla 35–45 mm long	***A. molinae* (Honduras)**
20	Corolla tube 5 mm wide at base or larger	***A. gentryi* (Costa Rica)**
–	Corolla tube less than 5 mm wide at the base	***A. breedlovei* (Mexico)**
21	Leaves 20–40 cm long × 5–15 cm wide; inflorescences on the main trunk and on the old branches; restricted to lowland forests (below 1000 m alt)	***A. kennedyae* (Honduras, Costa Rica and Panama)**
–	Leaves smaller 5–20 cm long × 1–5 cm wide, inflorescences on the old branches and among the foliage; growing in montane forests (between 900 m and 1500 m alt)	***A. silvicola* (Mexico and Guatemala)**
22	Scandent shrub; leaves 20 cm long or larger; inflorescences on the main trunk	***A. parviflora* (Panama)**
–	Small trees; leaves up to 15 cm long; inflorescences on old branches and among the foliage	***A. donnell-smithii* (Mexico and Guatemala)**

## Discussion

### Morphological groups and their distribution

The results presented here show that *Amphitecna* consists of several morphological groups (Figure 1). These groups do not necessarily represent lineages and, according to internal clustering validation (*Si* value), their stability should be tested with additional data. Nonetheless, the resulting morphological grouping recovered provides new insights into the understanding of relationships among species of *Amphitecna*.

Although little is known about the reproductive ecology of *Amphitecna*, the flower and fruits differences among groups are likely linked to their pollinators and seed dispersers. Most species have exposed inflorescences (terminal and cauliflorous), consisting of one-to several flowers with a transverse fold in the corolla throat fitting the *Crescentia*-type pollination syndrome, which includes bat-pollinated flowers (Gentry 1980, Fleming et al. 2009). However, hummingbirds and other birds also visit flowers of some *Amphitecna* species, such as *A. apiculata*, *A. latifolia*, and *A. sessilifolia* (Richardson 1984). On the other hand, the fleshy and indehiscent fruits (mostly mammalian-dispersed) are only found in *Amphitecna* and close relatives (Gentry 1980), showing considerable variation in fruit shape and surface.

The distribution of species within the various morphological groups seems to follow a geographical pattern. Cluster “A” (species with a transverse fold in the corolla throat) includes taxa that are distributed throughout Mesoamerica (from Mexico to Colombia) (Table 1). On the other hand, sub-groups within cluster “A” show variable distribution patterns. For example, the *A. molinae* group from cluster “A” has members in both regions of Mesoamerica (i.e., Northern Mesoamerica and Southern Mesoamerica; Table 1), with the *A. megalophylla* group endemic to the northern portions of Mesoamerica (from Mexico and Guatemala), and the *A. spathicalyx* group endemic to the southern portions of Mesoamerica (found in Panama exclusively). On the other hand, cluster “B” includes members of the *A. macrophylla* and *A. donnell-smithii* groups, occurring predominantly in northern Mesoamerica. Except from *A. parviflora* that is endemic to Costa Rica, the remaining six species of cluster “B” are found in Mexico and Guatemala exclusively. Finally, two species placed in the *Amphitecna steyermarkii* group occur in northern Mesoamerica (Mexico, Guatemala, and Belize), with a single species endemic to Costa Rica. Based on the above, northern Mesoamerica is not only the center of diversity of *Amphitecna*, but also the most diverse region morphologically (Table 1).

**Table 1.** Species currently recognized in *Amphitecna* (Bignoniaceae) and their respective geographical distribution. The species are ordered by the morphological groups recovered in the clustering analysis. *Amphitecna latifolia* (from the *A. molinae* group, cluster “A”) was excluded because it is the only broadly distributed species. NM = Northern Mesoamerica; SM = Southern Mesoamerica.

Species	Group	Cluster	Distribution
*Amphitecna costata* A.H.Gentry	*Amphitecna megalophylla* group	A	NM: Guatemala
*Amphitecna megalophylla* (J.D.Sm.) A.H.Gentry	*Amphitecna megalophylla* group	A	NM: Guatemala
*Amphitecna apiculata* A.H.Gentry	*Amphitecna molinae* group	A	NM: Mexico, Guatemala, Belize
*Amphitecna breedlovei* A.H.Gentry	*Amphitecna molinae* group	A	NM: Mexico, Guatemala, Belize
*Amphitecna fonceti* Ortiz-Rodr. & Gómez-Domínguez	*Amphitecna molinae* group	A	NM: Mexico
*Amphitecna gentryii* W.C.Burger	*Amphitecna molinae* group	A	SM: Costa Rica
*Amphitecna isthmica* (A.H.Gentry) A.H.Gentry	*Amphitecna molinae* group	A	SM: Costa Rica, Panama, Colombia
*Amphitecna kennedyae* (A.H.Gentry) A.H.Gentry	*Amphitecna molinae* group	A	SM: Honduras, Costa Rica, Panama, Colombia
*Amphitecna molinae* L.O.Williams	*Amphitecna molinae* group	A	SM: Honduras, Nicaragua
*Amphitecna sessilifolia* (Donn.Sm.) L.O.Williams	*Amphitecna molinae* group	A	SM: Costa Rica
*Amphitecna silvicola* L.O.Williams	*Amphitecna molinae* group	A	NM: Mexico, Guatemala
*Amphitecna spathicalyx* (A.H.Gentry) A.H.Gentry	*Amphitecna spathicalyx* group	A	SM: Panama
*Amphitecna donnell-smithii* (Sprague) L.O.Williams	*Amphitecna donnell-smithii*group	B	NM: Guatemala
*Amphitecna parviflora* A.H.Gentry	*Amphitecna donnell-smithii*group	B	SM: Costa Rica
*Amphitecna loreae* Ortíz-Rodr. & Burelo	*Amphitecna macrophylla* group	B	NM: Mexico
*Amphitecna macrophylla* Miers ex Baill.	*Amphitecna macrophylla* group	B	NM: Mexico
*Amphitecna montana* L.O.Williams	*Amphitecna macrophylla* group	B	NM: Mexico, Guatemala
*Amphitecna regalis* (Linden) A.H.Gentry	*Amphitecna macrophylla* group	B	NM: Mexico
*Amphitecna tuxtlensis* A.H.Gentry	*Amphitecna macrophylla* group	B	NM: Mexico
*Amphitecna haberi* A.H.Gentry	*Amphitecna steyermarkii* group	C	SM: Costa Rica
*Amphitecna lundellii* A.H.Gentry	*Amphitecna steyermarkii* group	C	NM: Guatemala, Belize
*Amphitecna steyermarkii* (A.H.Gentry) A.H.Gentry	*Amphitecna steyermarkii* group	C	NM: Mexico, Guatemala

### Taxonomic implications

*Amphitecna megalophylla* was first treated as a synonym of *A. macrophylla* by Seibert (1940), which was subsequently followed by Standley and Williams (1974), Nelson (2008), and the iPlants Project (http://powo.science.kew.org/taxon/11655-2). Although Gentry (1980) highlighted the morphological features that characterize *A. megalophylla*, the species has continued to be treated as a synonym.

The results presented here show that *Amphitecna megalophylla* and *A. macrophylla* are clearly distinct and are best treated as separate taxa (Table 2). *Amphitecna megalophylla* is part of the *A. megalophylla* group together with *A. costata*, both of which are placed within cluster “A” based on its multi-flowered inflorescences, buds rounded at apex, and long pedicellate corollas with a transverse fold in the throat (Figure 1). In contrast, *A. macrophylla* is placed within the *A. macrophylla* group (cluster “B”) along with two other giant-leaved species based on its inflorescences with 1 (rarely 2) short pedicellate flowers that lack a transverse fold in the corolla throat (radially symmetric). In addition, *A. macrophylla* is endemic to Veracruz (Mexico), while *A. megalophylla* is endemic to Guatemala (Gentry 1980).

**Table 2.** Comparison of diagnostic morphological features among the giant-leaved species of *Amphitecna* (Bignoniaceae).

Morphological features	*Amphitecna costata*	*Amphitecna macrophylla*	*Amphitecna megalophylla*	*Amphitecna regalis*
Habit	Branched tree	Pachycaul tree	Pachycaul tree	Pachycaul tree
Leaf long	Less than 60 cm	Less than 60 cm	up to 100 cm	up to 100 cm
Flowers per shoot	1 or 2	1-to-several	3-to-several	1-to-several
Pedicel length	up to 25 mm	up to 25 mm	up to 60 mm	up to 10 mm
Calyx length	up to 15 mm	up to 35 mm	up to 18 mm	up to 28 mm
Transverse fold in the throat of corolla	Present	Absent	Present	Absent
Corolla length	up to 40 mm	up to 50 mm	up to 40 mm	up to 60 mm
Fruit surface	Costate	Smooth	Costate	Not seen

Results from our cluster analyses suggest that *A. fonceti* is part of the *A. molinae* group (cluster “A”) along with *A. apiculata*, *A. latifolia*, and *A. sessilifolia*. Species within cluster “A” share multi-flowered inflorescences and flowers with a transverse fold in the throat, while showing several differences in their flower and fruit morphology (Table 3). Hence, *A. fonceti* is best treated as a separate taxon, which is described below and compared to other morphologically similar taxa.

**Table 3.** Comparison of diagnostic morphological features among *Amphitecna fonceti* (Bignoniaceae) and close relatives.

Morphological features	*Amphitecna fonceti*	*Amphitecna apiculata*	*Amphitecna latifolia*	*Amphitecna sessilifolia*
Leaf long	Up to 40 cm	Up to 40 cm	Up to 20 cm	Up to 30 cm
Leaf wide	Up to 13 cm	Up to 12 cm	Up to 11 cm	Up to 9 cm
Inflorescences	Mostly ramiflorous	Mostly terminal	Mostly terminal	Terminal
Pedicel length	Up to 60 mm	Up to 50 mm	Up to 40 mm	Up to 80 mm
Calyx length	up to 32 mm	up to 20 mm	up to 37 mm	up to 30 mm
Calyx surface	Strongly costate, pubescent and densely covered with lenticels-like white dots	Smooth and glabrous	Smooth and glabrous	Smooth and glabrous
Corolla shape	Funnelform	Tubular	Funnelform	Funnelform
Corolla length	up to 45 mm	up to 28 mm	up to 62 mm	up to 52 mm
Corolla mouth	23 mm diam.	10 mm diam.	24 mm diam.	16 mm diam.
Stamens	3, rarely 4	4	4	4
Stamen insertion	4–12 mm from base of the tube	10 mm from base of the tube	15–20 mm from base of the tube	13–18 mm from base of the tube
Style length	33–37 mm	?	45–50 mm	38–39 mm
Fruits shape	Elliptic	Elliptic	Globose	Elliptic
Fruits apex	Acute to short apiculate	Apiculate	Rounded	Apiculate
Habitat	Oak forest at c. 1500 m altitude	Lowland wet forest mostly below 500 m altitude	Restricted to coastal forest and mangrove	Montane wet forest mostly between 1300 and 2000 m alt

## Taxonomic treatment

### Amphitecna fonceti

Taxon classificationPlantaeLamialesBignoniaceae

Ortiz-Rodr. & Gómez-Domínguez sp. nov.

C5775C4C-7F9F-5033-A047-9DF69FF88878

urn:lsid:ipni.org:names:77214647-1

Figures 2 , 3

#### Type.

Mexico. Chiapas, Municipio de La Concordia, Área de Protección de Recursos Naturales La Fraylesca, Rancho “Pacayal” a 3 kilómetros del ejido Solo Dios,1441 m, 15°46'57.7"N, 92°59'04.6"W, 24 May 2020(fl, fr) *Gómez- Domínguez H. y Hernández-Burguete R. 3840* (holotype HEM; isotypes: MEXU, MO).

**Figure 2.**
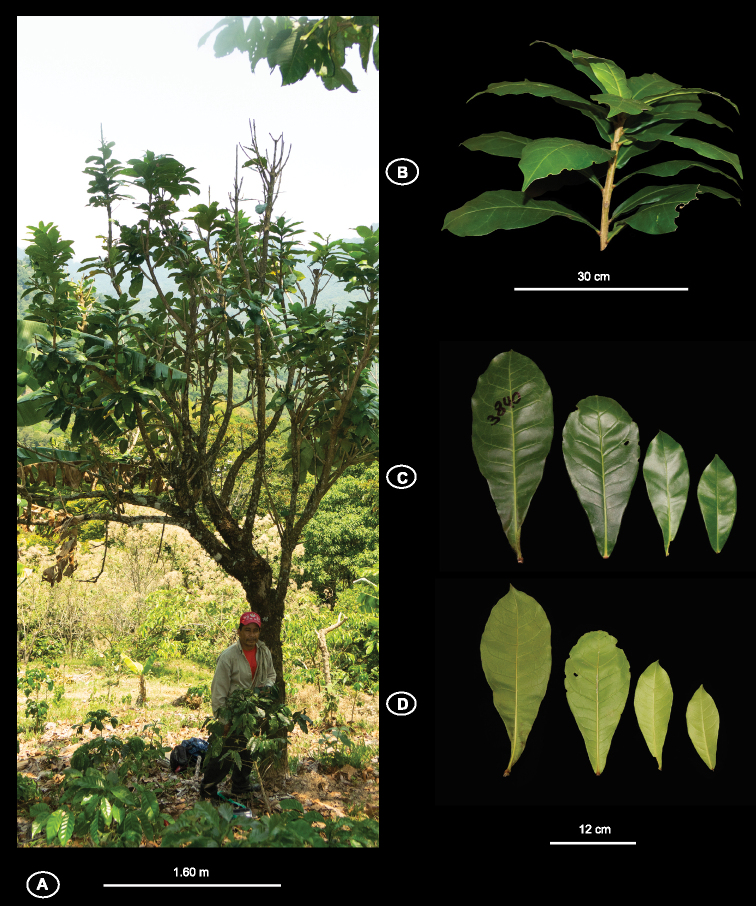
Vegetative features of *Amphitecna fonceti* sp. nov. **A** habit **B** phyllotaxy **C** adaxial side of leaf **D** abaxial side of leaf. Photographs by Hector Gómez Domínguez.

#### Diagnosis.

*Amphitecna fonceti* is distinguishable from the other species of *Amphitecna* by its ramiflorous inflorescences that bear multiple flowers per shoot, buds rounded at apex, large flowers with a transverse fold in the corolla throat, calyx surface pubescent and strongly costate, and fruits elliptic, apiculate at the apex. *Amphitecna fonceti* is morphologically similar to *A. apiculata* and *A. latifolia*, both of which occur in Mexico. However, *A. apiculata* differs by the small and tubular corollas, and by the calyx with a smooth and glabrous surface. *Amphitecna latifolia*, on the other hand, differs by the smaller leaves, smooth and glabrous calyx surface, and globose fruits with a rounded apex. The three species show different climatic preferences (Table 3).

#### Description.

Small to medium sized trees, 3–9 m alt., 6–25 cm DBH, the secondary branches terete. *Leaves* alternate-verticillate, clustered near the apex of branches, olive-green when dry, glabrous, coriaceous, 13–35 cm long × 6–13.2 cm wide, oblanceolate to obovate, short acuminate, acute to attenuate basis, midrib slightly raised on the upper surface, prominent on the lower surface; secondary veins 10–20 on each side, slightly raised above, prominent below; petiole shorter than 1 cm long, merging with attenuate leaf base, red wine *in vivo*. *Inflorescences* bearing three to six flowers (rarely with a single flower), borne on leafless portions of old branches, rarely terminal or along the main trunk (cauliflory), with a sour-odor; pedicels, outer side of buds, and calyces pubescent and densely covered with lenticel-like white dots. *Flowers* more or less erect, not pendant, pedicel 38–60 mm long; buds, rounded at apex; calyx campanulate, 25–32 mm long, coriaceous, evenly *2–3*-labiate, strongly costate, with 6–10 longitudinal ridges per lobe; corolla funnelform, with a transverse fold on throat between 22–27 mm from the base, pale green, 38–46 mm long × 20–23 mm wide at the tube mouth, the basal portion of the corolla funnel-shaped, 9–13 mm long, lobes more or less fused into a frilly-margined rim; androecium with stamens 3 or 4, included, inserted 4–12 mm from base of the tube, anther thecae divergent, 5–6 mm long, filaments 12–29 mm long, staminodes shorter than 20 mm long when present, inserted 3–6 mm from base of the tube; gynoecium with ovary ca. 8 mm long × ca. 4 mm wide, broadly elliptic, glandular-papillose, style 25–29 mm long, stigma bifurcate; disc annular-pulvinate, ca. 11 mm in diameter. *Fruits* elliptic, 110–180 mm long × 70–105 mm wide, acute to short acuminate at apex, rounded to short acuminate at the base.

**Figure 3.**
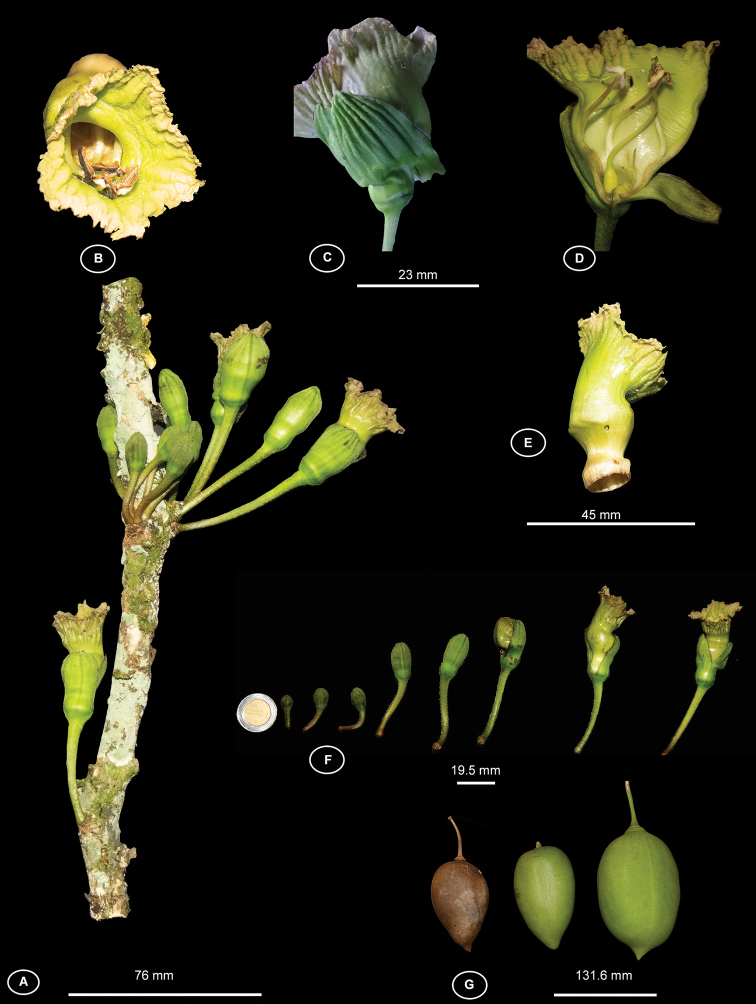
Reproductive features of *Amphitecna fonceti* sp. nov. **A** ramiflorous inflorescences with several flowers per shoot **B** corolla mouth **C** strongly costate calyx **D** corolla showing three stamens **E** corolla showing the transverse fold in the throat **F** flower developmental stages, from bud to anthesis **G** fruit shape variation. Photographs by Hector Gómez Domínguez.

#### Habitat and ecology.

This species is known only from the type locality in Chiapas, Mexico. The species inhabits areas with sedimentary soils, mostly formed by sandstones with a thin layer of organic matter, mostly within altered remnants of oak and pine-oak forest. The species with which it coexists are *Quercus rugosa* Née, *Inga vera* Willd, *Damburneya coriacea* (Sw.) Trofimov & Rohwer, *Eugenia capuli* (Schltdl. & Cham.) Hook. & Arn., *Trema micrantha* (L.) Blume, *Cecropia obtusifolia* Bertol., and *Coffea arabica* L.

#### Phenology.

Specimens were collected in full bloom or with ripe fruit in April and May. Flower buds were observed in March and ripe fruits in June.

#### Etymology.

The speciﬁc epithet honors FONCET (Fondo de Conservación El Triunfo, A.C.), in recognition of 18 years of funding dedicated to conservation projects in natural protected areas within the Sierra Madre de Chiapas, Mexico.

#### Conservation status.

According to the IUCN (2019), this species is considered as Critically Endangered [CR B1ab (iii)]. Its area of occupancy (AOO) is 8.0 km^2^ and the extent of occurrence (EOO) is 0.154 km^2^, showing a restricted distribution. Although the new species is distributed within a protected natural area, the oak, pine-oak forest at the type locality is seriously fragmented, with only small remnants persisting. *Amphitecna fonceti* is rare, with only 12 individuals being known to date.

#### Uses.

The indigenous community where *A. fonceti* is found uses the fruits to treat respiratory diseases. The seeds of ripe fruits are extracted and soaked in a bottle of tequila for a week, after which a small glass is drunk in the morning to treat asthma. For whooping cough, two tablespoons of honey and almond oil are poured into the fruit after the removal of the fruit tip. The fruit is then cooked in water bath and its interior used as syrup. Its medicinal use likely helps the maintenance of this species within local coffee plantations.

#### Additional specimens examined.

Mexico. Chiapas, La Concordia: Área de Protección de Recursos Naturales, La Fraylesca; Rancho Pacayal a 3 kilómetros del ejido Solo Dios, 15°46'54.9"N, 92°59'04.8"W, 1359 m., 24 de Mayo de 2020., *Gómez- Domínguez, H.* and *Hernández-Burguete, R. 3841* (HEM); same locality, *Gómez- Domínguez, H.* and *Hernández-Burguete, R. 3842* (HEM); *Gómez- Domínguez, H., Velazco Espino, D.* and *Hernández-Burguete, R. 3841* (XAL).

#### Notes.

In addition to *A. apiculata* and *A. latifolia*, *A. fonceti* can also be confused with *A. sessilifolia*, another species from the *A. molinae* group. However, *A. sessilifolia* (endemic to Costa Rica) shows terminal flowers, larger corollas, stamens inserted 13–18 mm from base of the corolla tube, larger pistils, smooth and glabrous calyces (Gentry 1980, Table 3). *Amphitecna sessilifolia* has been incorrectly reported to Mexico (Martínez-Meléndez et al. 2017) based on misidentified specimens of *A. breedlovei* (e.g., Faustino Miranda 6916, MEXU-67682), *A. latifolia* (e.g., G. Martínez C. 2294, MEXU-733205), and *A. tuxtlensis* (e.g., J.I. Calzada 1457, MEXU-309621).

##### *Amphitecna megalophylla* resurrected

Our results indicate that *A. megalophylla* is best treated as a separate taxon that can be identified by the following features: pachycaul trees, with leaves up to 1 m long, multi-flowered inflorescences, cauliflorous and long-pedicellate flowers with a transverse fold in the corolla throat, and fruits with costate/angulate surfaces. The following species is thus treated as an accepted taxon here:

### Amphitecna megalophylla

Taxon classificationPlantaeLamialesBignoniaceae

(Donn. Sm.) A.H. Gentry

383F51F2-B111-534A-946D-20F387483195

Neotuerckheimia megalophylla Donn. Sm., Bot. Gaz. (Crawfordsville) 47: 258, f.l. 1909. Basionym.

#### Distribution.

Guatemala (endemic).

#### Specimens examined.

Guatemala. Alta Verapaz, Coban: 1350 m, *Türckheim H. von II 2278* (isosyntype, M).

## Supplementary Material

XML Treatment for Amphitecna fonceti

XML Treatment for Amphitecna megalophylla

## Supplementary materials

Supplementary material 1 Figure S1. Cophenetic correlation between clustering results from eight different linkage algorithms and Gower similarity index. Data type graphical data type https://binary.pensoft.net/file/501138This dataset is made available under the Open Database License (http://opendatacommons.org/licenses/odbl/1.0/). The Open Database License (ODbL) is a license agreement intended to allow users to freely share, modify, and use this Dataset while maintaining this same freedom for others, provided that the original source and author(s) are credited.Héctor Gómez-Domínguez, Andrés Ernesto Ortiz-Rodríguez, Delfilia Velasco-Espino, Rene Hernández-Burguete

Supplementary material 2 Morphological dataset Data type morphological Explanation note Flower and leaves characteristics of 24 species of *Amphitecna*. https://binary.pensoft.net/file/501139This dataset is made available under the Open Database License (http://opendatacommons.org/licenses/odbl/1.0/). The Open Database License (ODbL) is a license agreement intended to allow users to freely share, modify, and use this Dataset while maintaining this same freedom for others, provided that the original source and author(s) are credited.Héctor Gómez-Domínguez, Andrés Ernesto Ortiz-Rodríguez, Delfilia Velasco-Espino, Rene Hernández-Burguete
