# Supplementary figures and images for: Taxonomic updates in Amphitecna (Bignoniaceae): A new Mexican species and the re-establishment of the giant-leaved A. megalophylla
Source: PhytoKeys. 2021 Jan 25;171:75–90. doi: 10.3897/phytokeys.171.55397 (PMC7851107; doi:10.3897/phytokeys.171.55397)

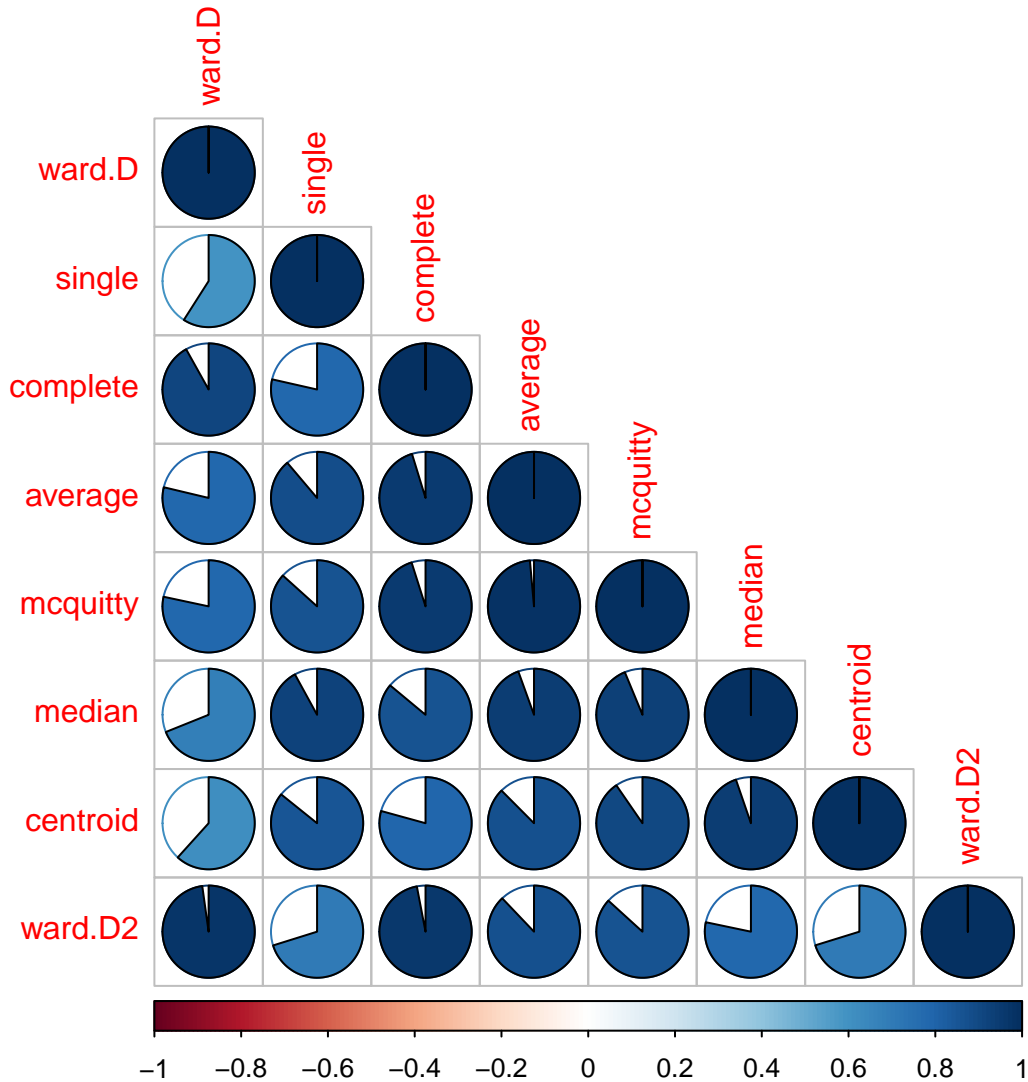

Supplement: Supplementary material 1 — Figure S1. Cophenetic correlation between clustering results from eight different linkage algorithms and Gower similarity index. [file phytokeys-171-075-s001.pdf]
